# Supplementary material for: Adhesive, Transparent Tannic Acid@ Sulfonated Lignin-PAM Ionic Conductive Hydrogel Electrode with Anti-UV, Antibacterial and Mild Antioxidant Function
Source: Materials (Basel). 2019 Dec 10;12(24):4135. doi: 10.3390/ma12244135 (PMC6947526; doi:10.3390/ma12244135)
Supplement: Supplementary file 1 [file materials-12-04135-s001.pdf]

materials

Supplementary

Adhesive, Transparent Tannic Acid@ Sulfonated Lignin-PAM Ionic Conductive Hydrogel Electrode with Anti-UV, Antibacterial and Mild Antioxidant Function

Qinhua Wang, Hui Zhang, Xiaofeng Pan, Xiaojuan Ma *, Shilin Cao * and Yonghao Ni *

College of Material Engineering, Fujian Agriculture and Forestry University, Fuzhou 350002, China; fafuqhw@163.com (Q.W.); 17862266728@163.com (H.Z.); 1171092015@fafu.edu.cn (X.P.)

* Correspondence: 1212juanjuan@163.com (X.M.); scutcsl@163.com (S.C.); yonghao@unb.ca (Y.N.)

Received: 29 October 2019; Accepted: 9 December 2019; Published: 10 December 2019

1. Preparation of Ca^{2+}-TA@SL-PAM Hydrogel

The Ca^{2+}-tannic acid@sulfonated lignin-polyacrylamide (TA@SL-PAM) hydrogel was prepared via a four-step process. First, SL was prepared via lignin sulfonation. The detailed sulfonation process was described in our previous study [1]. After that, TA was added into the SL dispersion and stirred for 10 min. The pH of the mixed suspension was adjusted to 8 using Tris buffer (1 M), the resultant suspension was magnetically stirred for 8 h at room temperature. Next, the calcium chloride ($CaCl_2$) powder was added into the suspension and stirred well. Finally, acrylamide (AM), ammonium persulfate (APS), N, N′-Methylene bisacrylamide (BIS), and tetramethylethylenediamine (TMEDA) were added to the Ca^{2+}-TA@SL suspension in an ice bath. After 10 min stirring, AM were polymerized and crosslinked to form the Ca^{2+}-TA@SL-PAM hydrogel.

Table S1. Detailed composition of various hydrogels.

Samples	SL (g)	TA/SL	Calcium Chloride (g)	AM (g)	APS (g)	BIS (g)	TMEDA (μL)	Water (mL)
PAM	0	0	0	2.5	0.25	0.003	10	10
PAM-SL	0.25	0	0.111	2.5	0.25	0.003	10	10
PAM-1	0.25	0.1	0.111	2.5	0.25	0.003	10	10
PAM-2	0.25	0.2	0.111	2.5	0.25	0.003	10	10
PAM-3	0.25	0.3	0.111	2.5	0.25	0.003	10	10
PAM-4	0.25	0.4	0.111	2.5	0.25	0.003	10	10
PAM-5	0.25	1	0.111	2.5	0.25	0.003	10	10

2. Antioxidant Assay

The antioxidant efficiency of hydrogels was evaluated by scavenging stable 1, 1-diphenyl-2-picryl-hydrazyl (DPPH) radicals. First, the hydrogel was cut into homogenates using a tissue grinder, then PAM-5 hydrogel samples (24 mg, 36 mg, and 48 mg) were dispersed in 3.0 mL DPPH/ethanol solution (100 μM), respectively, and the mixture was stirred and kept in the dark at room temperature for 30 min. Finally, the absorbance at 517 nm was measured with an ultraviolet spectrophotometer. DPPH radical scavenging activity was calculated as the following formula:

$$\text{DPPH scavenging activity} = \left(1 - \frac{A_S}{A_O}\right) \times 100\% \tag{1}$$

where A_o is the absorbance of DPPH solution and A_s is the absorbance of samples mixed with DPPH solution. Each sample was carried out in triplicate.

3. Antibacterial Assay

Staphylococcus aureus (*S. aureus*, ATCC 29213) were employed to test the hydrogel (PAM, PAM-SL, and PAM-5) surface antibacterial activity. In brief, one single colony was selected and placed into 10 mL LB solution at 37 °C and cultured for 18 h. (The concentration of bacterial solution was controlled to be 10^{5-6}CFU/mL). Then, 0.5 g hydrogel was placed in 5 mL LB culture medium, 100 μL bacterial suspension was inoculated on the gel sample, and the concentration of bacteria in the final sample was 10^{3-4} CFU/mL. The inoculated materials were placed in a 37 °C incubator and incubated at 130 rpm for 18 h. After 0.1% gradient dilution of peptone water, 0.1 mL was applied on the LB plate. The coated plate was placed in a 37 °C incubator for 24 h, and then taken out and counted.

$$\text{DPPH scavenging activity} = \left(1 - \frac{A_S}{A_O}\right) \times 100\% \quad (2)$$

where A and B are the number of bacterial colonies surviving in the test sample and control group, respectively.

References

1. Wang, Q.; Pan, X.; Lin, C.; Lin, D.; Ni, Y.; Chen, L.; Huang, L.; Cao, S.; Ma, X. Biocompatible, self-wrinkled, antifreezing and stretchable hydrogel-based wearable sensor with PEDOT: sulfonated lignin as conductive materials. *Chem. Eng. J.* **2019**, *370*, 1039–1047.

© 2019 by the authors. Submitted for possible open access publication under the terms and conditions of the Creative Commons Attribution (CC BY) license (http://creativecommons.org/licenses/by/4.0/).
